# Optimal MRI sequence for predicting extraprostatic extension in prostate cancer: comparison of LCC and MaxRADD measurements

**DOI:** 10.1007/s00261-026-05615-w

**Published:** 2026-06-24

**Authors:** Gorun Ilanjian, Jade Matthey, Fabio Porões, Massimo Valerio, Adrien Richemond, Julien Dagher, Arnas Rakauskas, Clarisse Dromain, Mario Jreige, Naïk Vietti-Violi

**Affiliations:** 1 Radiology department, University Hospital of Lausanne, Lausanne, Switzerland; 2 Urology Department, University Hospital of Geneva, Geneva, Switzerland; 3 UNIL, University of Lausanne, Lausanne, Switzerland; 4 Pathology Department, University Hospital of Lausanne, Lausanne, Switzerland; 5 Urology Department, University Hospital of Lausanne, Lausanne, Switzerland; 6 Nuclear Medecine Department, University Hospital of Lausanne, Lausanne, Switzerland

**Keywords:** Dynamic contrast-enhanced, Extra-prostatic extension, Length capsular contact, Maximal lesion radial distance, Multiparametric MRI, Prostate cancer

## Abstract

**Objectives:**

Accurate preoperative identification of extraprostatic extension (EPE) in prostate cancer (PCa) is important for treatment strategies. This retrospective study aimed to determine which MRI sequence provides the most accurate measurement of length of capsular contact (LCC) and maximum lesion radial distance (MaxRADD) for predicting EPE.

**Methods:**

We included 63 patients who underwent radical prostatectomy for biopsy-confirmed PCa and had preoperative multiparametric MRI (mpMRI) from 2018 to 2020. Two radiologists measured LCC and MaxRADD on T2-weighted imaging (T2WI), diffusion-weighted imaging (DWI), apparent diffusion coefficient (ADC) maps, and dynamic contrast-enhanced (DCE) sequences. LCC was evaluated using 10 mm and 15 mm thresholds based on Prostate Imaging-Reporting and Data System (PI-RADS) and European Society of Urogenital Radiology (ESUR) guidelines. Histopathology was the reference standard. Sensitivity, specificity, and area under the receiver operating characteristic curve (AUC) were calculated.

**Results:**

63 patients with 105 evaluable lesions were included. Significant differences were observed between the 10 mm and 15 mm LCC thresholds for predicting EPE. At 10 mm, DCE demonstrated the highest sensitivity (90.1%) and a 80.7% specificity. At 15 mm, DCE achieved higher specificity (94.7%) with 85% sensitivity. ROC analysis identified 14 mm on DCE as the optimal LCC threshold, reaching 90% sensitivity, 96.6% specificity (AUC:0.93). MaxRADD showed variable diagnostic performance, with the highest AUC on DCE (0.83), T2WI (0.80), DWI (0.78), and ADC (0.74), without significant differences. Combining LCC and MaxRADD slightly improved AUC (0.96 vs. 0.93 for LCC alone, *p* = 0.270). MaxRADD performed best on DCE at an 11 mm threshold (sensitivity 65%, specificity 88.5%).

**Conclusion:**

DCE provides the highest diagnostic performance for predicting EPE using LCC measurements, with a 14 mm LCC and 11 mm MaxRADD threshold yielding the best overall accuracy. Combining LCC and MaxRADD numerically increased AUC (0.96 vs. 0.93), however without significant differences in EPE prediction (*p* = 0.270).

## Introduction

Accurate local staging of prostate cancer (PCa) is essential for prognosis assessment and treatment planning [1, 2].

According to EAU and AUA guidelines, management strategies vary depending on disease stage and patient characteristics, ranging from active surveillance for low-risk disease to radiotherapy, focal therapies, such as experimental High Intensity Focused Ultrasound (HIFU) [3], systemic treatments, or radical prostatectomy for intermediate-, high-risk, and locally advanced tumors, including cases with extraprostatic extension (EPE) [4, 5].

Patients with an ISUP grade group ≥ 2 combined with EPE, face a high risk of progression, which can reach up to 50% within five years [2, 6]. The accuracy of mpMRI in predicting EPE varies across studies, with sensitivity around 57% (95% confidence interval [CI], 0.49–0.64) and average specificity approximately 91% (95% CI 0.88–0.93) [7]. Identifying EPE is essential for guiding surgery, as it may require abandoning nerve-sparing approaches to ensure oncological safety [8]. Recent studies show that EPE is found in approximately 18% to 32% of radical prostatectomy specimens [9, 10].

The measurement of the Length of Capsular Contact (LCC) is a quantitative metric that has shown to improve the accuracy of predicting EPE [11, 12]. A recent meta-analysis showed that the probability of EPE on histopathological analysis increases proportionally with the LCC [13]. However, there is a divergence in the recommended thresholds for this measurement: Prostate Imaging-Reporting and Data System (PI-RADS) suggests that an LCC greater than 10 mm predicts EPE [14], while the European Society for Urogenital Radiology (ESUR) proposes a threshold of 15 mm [15]. Additionally, while the ESUR and certain studies advocate performing LCC measurement on T2-weighted imaging (T2WI) [15], PI-RADS does not specify which sequence should be used for this measurement [16].

Moreover, a recent study introduced the maximum lesion radial distance (MaxRADD), a novel MRI quantitative parameter measuring tumor diameter perpendicular to the capsule for predicting EPE, and showed similar diagnostic accuracy to LCC (AUC 0.92 vs. 0.91) with a more balanced sensitivity and specificity, suggesting it may be a robust and reproducible tool for improving preoperative assessment in PCa [17].

T2WI and diffusion-weighted imaging (DWI) are the key components of prostate MRI, each providing complementary information. T2WI offers excellent anatomical resolution and is essential for evaluating prostatic zonal anatomy and capsular morphology, but its ability to detect subtle EPE can be limited. DWI improves lesion detection and tumor characterization by reflecting tissue cellularity, enhancing the identification of clinically significant cancer; however, it is more prone to artifacts and provides less precise anatomical delineation.

We hypothesized that DCE may provide superior performance for predicting EPE compared with T2WI and DWI. This hypothesis is based on the ability of DCE to reflect tumor vascularity and microvascular permeability, which may be associated with tumor angiogenesis and extracapsular tumor spread.

This study aimed to identify the mpMRI sequence with the highest diagnostic performance for predicting EPE using prostatectomy specimens as the reference standard and to determine the optimal LCC threshold for this prediction. The second objective was to assess the predictive value of the MaxRADD, for EPE detection. Specifically, we aimed to determine whether MaxRADD provides greater accuracy than LCC and to evaluate whether combining MaxRADD with LCC improves the prediction of EPE compared to either parameter alone.

## Materials and methods

### Study design

This is a retrospective, observational and single-center study was approved by the local ethical committee (CER-VD 2020 − 00396) which waived the need for signed informed consent. Consecutive patients who underwent radical prostatectomy from May 2018 to February 2020 for biopsy proven PCa in our institution with preoperative prostate MRI were included.

Patients with previous treatment for PCa (radiotherapy, hormone therapy and/or focal therapy), with incomplete or low image quality (PI-QUAL V1 < 3), with non–clinically significant PCa (ISUP grade < 2 or pathologic volume < 0.5 ml) were excluded. The final population included 63 patients (64 years, IQR: 60–69, Table 1). The average time between the prostate MRI and the radical prostatectomy was 5.2 months.

**Table 1:** General patients and lesions characteristics

Variable	Total population (*n* = 63 patients; *n* = 105 lesions)	EPE-positive patients (*n* = 19)	EPE-negative patients (*n* = 44)	*p*-value (patient-level)	EPE-positive lesions (*n* = 20)	EPE-negative lesions (*n* = 85)	*p*-value (lesion-level)
	Median (IQR) or *n* (%)	Median (IQR) or *n* (%)	Median (IQR) or *n* (%)		Median (IQR) or *n* (%)	Median (IQR) or *n* (%)	
Patient-level characteristics							
Median age (years, IQR)	64.5 (60.2–67.5)	–	–	–	–	–	–
Median PSA (ng/mL, IQR)	8.5 (6.3–13.5)	9.4 (6.5–14.1)	7.0 (5.3–10.2)	0.105	8.6 (6.6–13.4)	7.0 (5.4–10.5)	0.101
MRI median prostatic volume (mL, IQR)	41.8 (32.4–60.0)	39.2 (35.5–43.9)	43.6 (31.2–62.2)	0.419	39.1 (36.5–43.6)	44.5 (32.4–62.7)	0.151
PSA density (ng/mL², IQR)	0.20 (0.12–0.29)	0.24 (0.17–0.38)	0.16 (0.11–0.27)	**0.042**	0.22 (0.18–0.38)	0.16 (0.11–0.24)	**0.017**
Lesion characteristics							
Number of lesions (%)	105	27 (26%)	78 (74%)	–	20 (19%)	85 (81%)	–
Mean lesion volume on pathology (mL, mean ± SD)	5.8 ± 7.9	–	–	–	9.7 ± 10.9	2.7 ± 5.5	< 0.001
Peripheral zone, n (%)	78 (74%)	20 (74%)	58 (74%)	1.000	15 (75%)	63 (74%)	1.000
Transitional zone, n (%)	27 (26%)	7 (26%)	20 (26%)		5 (25%)	22 (26%)	
ISUP grade group — final pathology, n (%)							
Grade group 1	17 (16%)	–	–		0 (0%)	17 (20%)	**< 0.001**
Grade group 2	52 (49.5%)	–	–		6 (30%)	46 (54%)	
Grade group 3	27 (26%)	–	–		7 (35%)	20 (24%)	
Grade group 4	1 (0.5%)	–	–		1 (5%)	0 (0%)	
Grade group 5	8 (8%)	–	–		6 (30%)	2 (2%)	

Bold p-values indicate statistical significance (*p* < 0.05)

*ADC* Apparent Diffusion Coefficient, *DCE* Dynamic Contrast Enhancement, *DWI* Diffusion Weighting Imaging, *EPE* Extra-prostatic extension, *IQR* Interquartile Range, *ISUP* International Society of Urological Pathology, *MRI*, magnetic resonance imaging, *PSA* Prostate-specific antigen, *T2WI* T2 Weighted-Imaging.

A patient is classified as EPE-positive if at least one lesion shows EPE on final pathology. Continuous variables: Mann-Whitney U test. Categorical variables: chi-square test

### MRI acquisition

All patients underwent 3 T prostate MRI (Siemens Healthineers, Skyra or Prisma) following ESUR technical recommendations for sequences and parameters. The MRI protocol section has therefore been expanded to include the main acquisition parameters for each sequence. Briefly, DWI was acquired using b-values of 50 and 1000 s/mm², with an additional calculated b-value image at 2000 s/mm² (TR 4400 ms, TE 65 ms). DCE imaging was performed using a 3D T1-weighted VIBE sequence (TR 4.9 ms, TE 1.77 ms, flip angle 14°) with 23 dynamic acquisitions and a temporal resolution of 8 s per acquisition. Gadolinium-based contrast agent was administered at a dose of 0.2 cc/kg with an injection rate of 2 cc/s, followed by a 15 cc saline flush. All sequences were acquired with T2WI images obtained in three orthogonal planes at 3 mm slice thickness, DWI using a ZOOMit reduced field-of-view technique at 3.5 mm, and DCE imaging acquired with a 3D T1-weighted VIBE sequence at 3.5 mm slice thickness, all compliant with PI-RADS v2.1 technical specifications.

### Image analysis

**Fig. 1:**
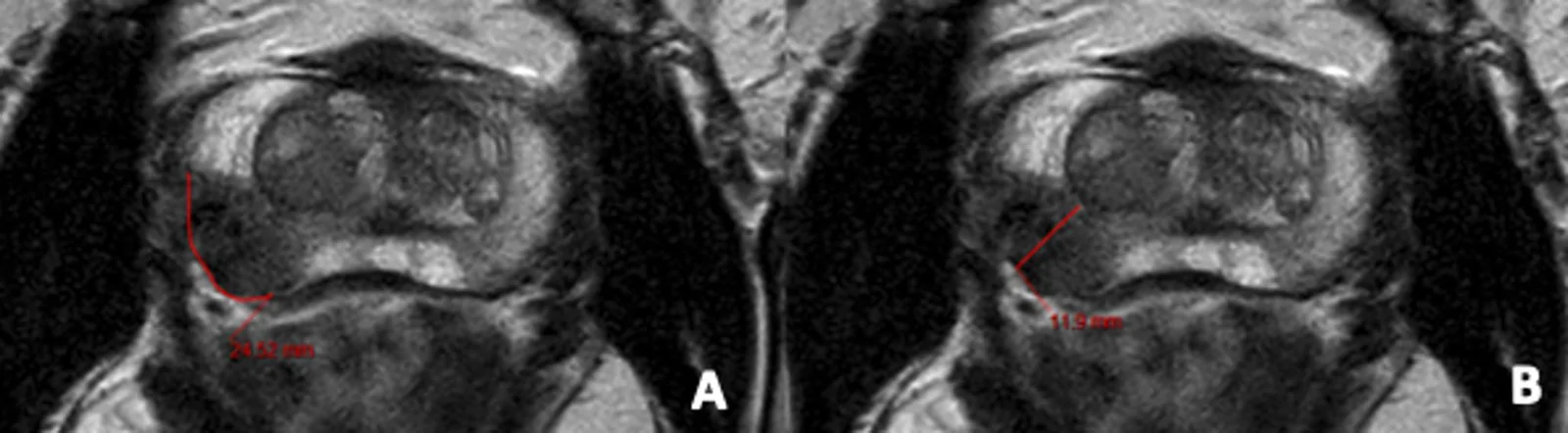
Illustration of prostate cancer measurements on MRI. LCC (**A**) curvilinear maximal tumor contact with the prostatic capsule. MaxRADD (**B**) maximum radial distance of the lesion, perpendicular to the capsule; LCC Length of Capsular Contact; MaxRADD Maximum lesion Radial Distance

**Fig. 2:**
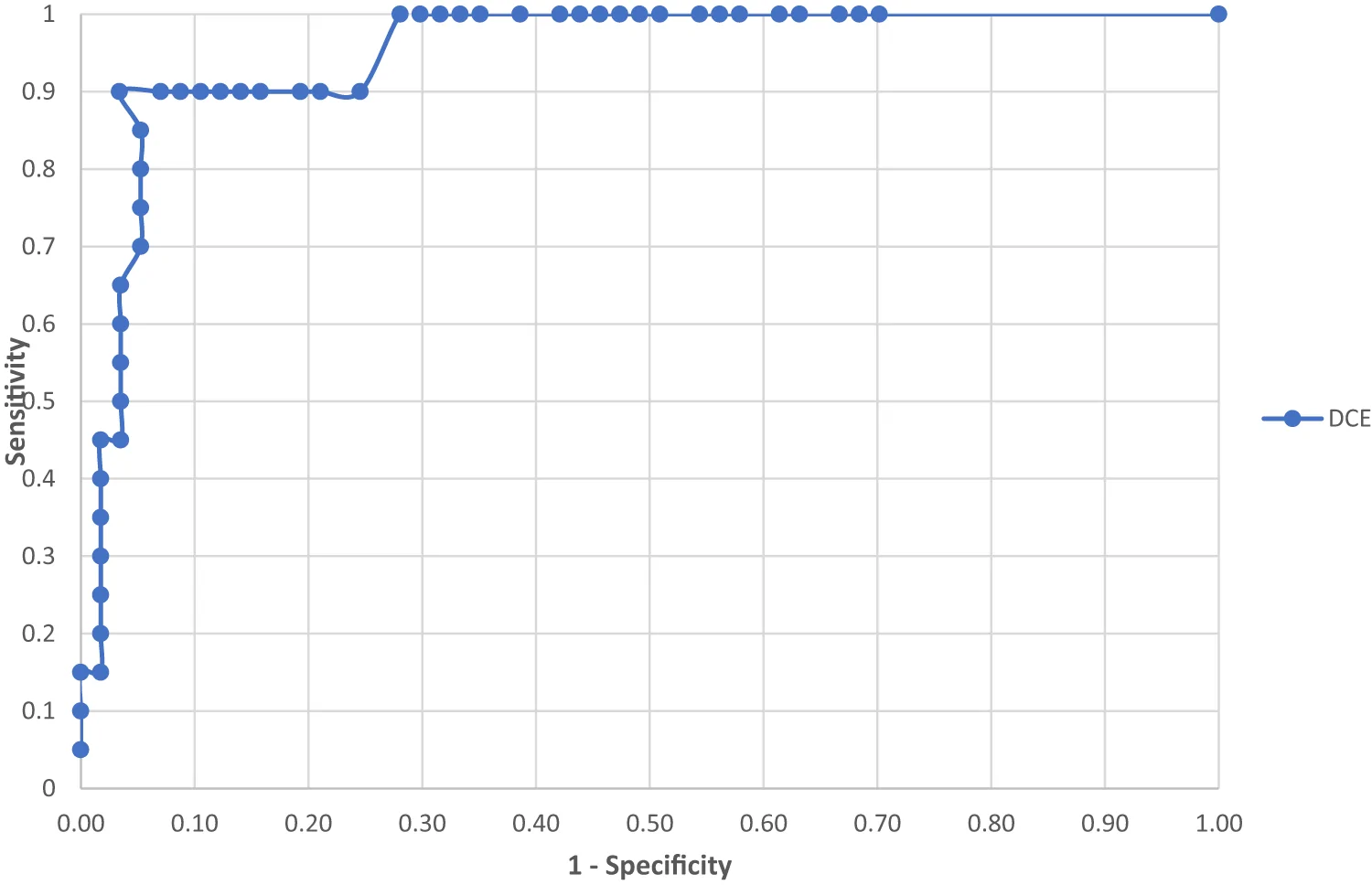
AUC curve for optimal thresholding DCE optimized for sensitivity and specificity. Each point on the curve represents the sensitivity and specificity pair for a given LCC threshold, ranging from 0 mm to the maximum observed value, with a threshold of 14 mm yielding a sensitivity of 90% and a specificity of 96.6%, representing the highest AUC

**Fig. 3:**
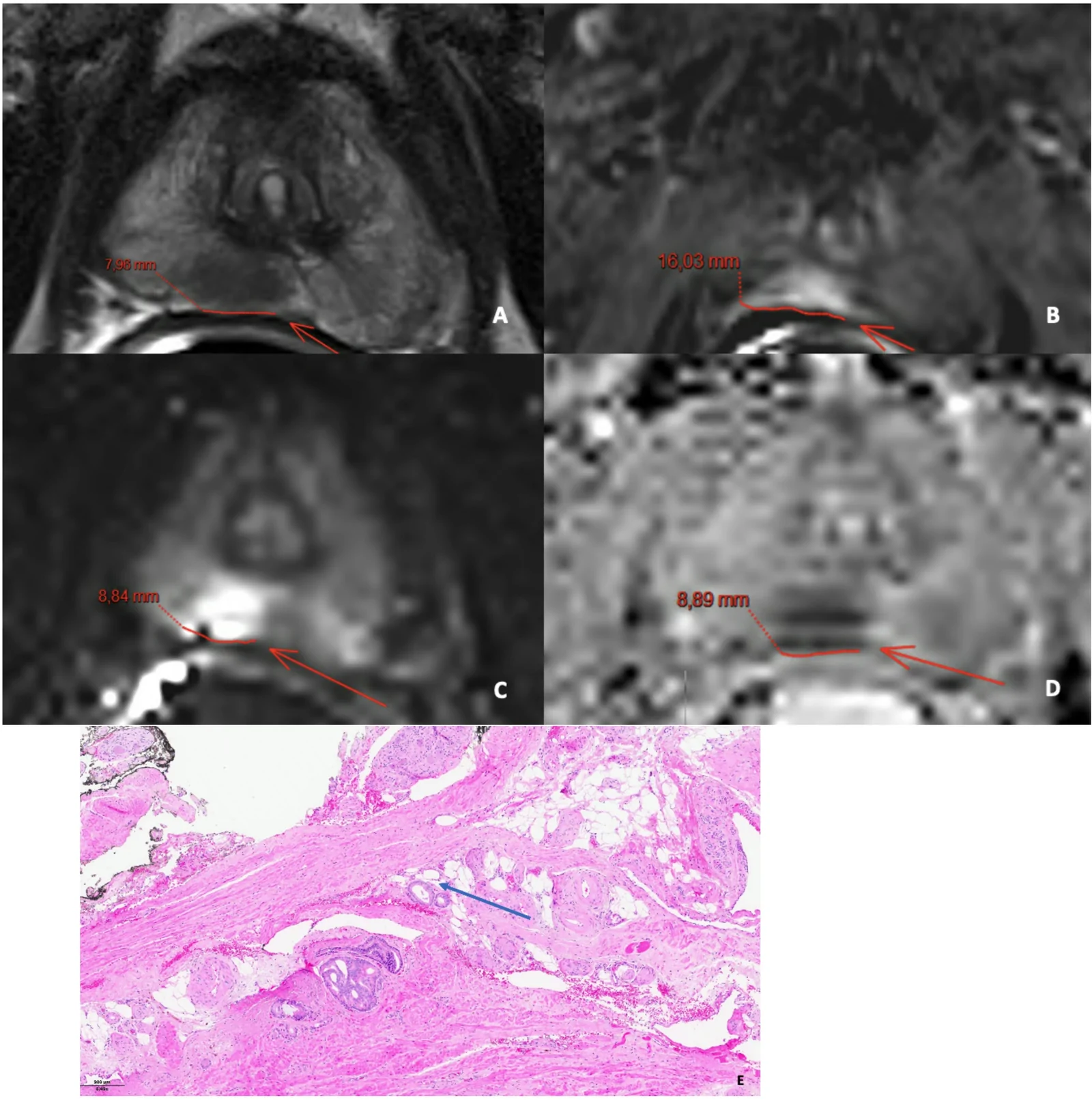
T2WI (**A**), DWI (**C**), ADC map (**D**), show a capsular contact < 10 mm but > 15 mm on DCE (**B**), represented with red arrows. EPE is positive on operative specimen (**E**): the blue arrow on pathology indicates tumoral glandular proliferation extending beyond the prostatic capsule into the periprostatic fat, consistent with EPE; ADC Apparent Diffusion Coefficient; DCE Dynamic Contrast Enhancement; DWI Diffusion Weighting Imaging; EPE Extra-Prostatic Extension; T2WI T2 Weighted-Imaging

In patients with multiple suspicious lesions and in accordance with PI-RADS recommendations, up to four lesions per patient could be considered. However, in practice, no patient in our cohort presented with more than three lesions simultaneously associated with EPE on final pathology. Lesions were selected by prioritizing the index lesion, defined as the lesion with the highest PI-RADS category and the greatest capsular contact. Lesions were first identified on DWI and T2WI as the anatomical reference sequence and subsequently matched across the corresponding ADC and DCE sequences. LCC measurements were then performed independently on each MRI sequence for the same index lesion, based on visual correspondence across sequences. For each lesion exhibiting capsular contact, curvilinear LCC was manually delineated and measured on each MRI sequence (T2WI, DWI, ADC maps, and DCE) using Mint Lesion^™^ software. In addition, the MaxRADD [17], defined as the short-axis thickness of the lesion measured orthogonally to the capsule, was measured in consensus (Fig. 1). For DCE imaging, capsular contact measurements were performed on subtraction images from the phase in which the lesion was most clearly visible. Measurements were performed in consensus by two radiologists with 2 and 12 years of experience in prostate MRI. No dedicated image co-registration was performed. Transition zone (TZ) lesions were evaluated according to their anatomical relationship with the prostatic capsule on multiparametric MRI. Only lesions demonstrating a clear interface with the capsule or extending toward the anterior fibromuscular stroma were included for quantitative analysis. LCC and MaxRADD measurements were performed on all MRI sequences in a complementary and interdependent manner, with each sequence (T2WI, DWI/ADC, and DCE when available) contributing to the overall assessment of capsular contact and lesion extent (See Fig. 2 and 3).

### Pathological analysis

Pathological analysis was performed using standard hematoxylin-eosin staining by two experienced pathologists with 8 and over 15 years of expertise in prostate pathology. In consensus, each lesion was first histologically confirmed. Radical prostatectomy specimens were entirely embedded and processed according to ISUP recommendations for specimen handling, although whole-mount histopathology sections were not used. Histopathological evaluation included precise annotation of the location of EPE relative to the tumor and prostate anatomy (slice number, anterior versus posterior location, left versus right side, and base versus apex), allowing MRI–pathology correlation. Pathologists were blinded to MRI findings during histopathological assessment.

### Statistical analysis

The LCC was evaluated as a radiological biomarker for predicting EPE, using 10 mm and 15 mm thresholds, in accordance with PI-RADS and ESUR recommendations, respectively.

Diagnostic performance was assessed for each MRI sequence (T2WI, DWI, ADC, and DCE) using sensitivity, specificity, and the area under the curve (AUC). Receiver operating characteristic (ROC) curve analysis was performed to evaluate the discriminative performance of LCC across sequences and thresholds.

MaxRADD was evaluated as an additional quantitative predictor of EPE. ROC analysis was performed for MaxRADD on each MRI sequence, and its diagnostic performance was compared with that of LCC. We then evaluated the optimal threshold of MaxRADD for EPE prediction across different MRI sequences and identified the sequence providing the highest diagnostic performance.

A combined model including LCC and MaxRADD was also explored using logistic regression to assess whether the combination improved the prediction of EPE compared with either parameter alone.

95% confidence intervals (95% CI) were calculated for sensitivity, specificity, and AUC values. Differences in AUC between MRI sequences were assessed using pairwise comparisons of ROC curves. To reduce the risk of overfitting related to ROC-derived threshold optimization, internal validation of the continuous DCE-based LCC model was additionally performed using bootstrap resampling with 1000 replications. A two-sided p-value < 0.05 was considered statistically significant.

All statistical analyses were performed using Stata software (version 18.0, StataCorp LLC, College Station, TX, USA).

## Results

A total of 63 men were included in the study, with a median PSA of 8.5 ng/mL (IQR 6.3–13.5). The mean lesion volume on pathology was 5.8 ± 7.9 mL, with 78 lesions (74%) located in the peripheral zone and 27 (26%) in the transitional zone. Across these patients, 107 prostate cancer lesions were identified on MRI and correlated with pathological analysis. Two lesions were excluded due to incomplete imaging data, resulting in 105 evaluable lesions. Of the 63 patients, 19 (30%) were EPE-positive and 44 (70%) EPE-negative, corresponding to 20 EPE-positive and 85 EPE-negative lesions at the lesion level. PSA density was significantly higher in EPE-positive patients compared to EPE-negative patients (0.24 [0.17–0.38] vs. 0.16 [0.11–0.27] ng/mL², *p* = 0.042), confirmed at the lesion level (0.22 [0.18–0.38] vs. 0.16 [0.11–0.24] ng/mL², *p* = 0.017). ISUP grade group distribution differed significantly between EPE-positive and EPE-negative lesions (*p* < 0.001), with no grade group 1 tumors among EPE-positive lesions versus 20% in the EPE-negative group, and a predominance of higher-grade lesions (grade groups 3–5) in the EPE-positive group (70% vs. 25%) (Table 1).

### Optimal sequence to predict EPE

Among the two tested thresholds, the best overall diagnostic performance was achieved at a 14 mm cutoff using the DCE sequence. At this threshold, DCE reached the highest combination of sensitivity and specificity: 90% (69.9–97.2) and 96.6% (90.3–98.8), respectively, with an AUC of 0.93 (95% CI 0.86–1.00), significantly outperforming all other sequences (Table 2; Figs. 2 and 3). Bootstrap internal validation of the continuous DCE-based LCC model using 1000 replications demonstrated stable diagnostic performance, with an internally validated AUC of 0.93 (95% CI 0.86–1.00), supporting the robustness of the identified 14 mm threshold.

**Table 2:** Sensitivity, specificity and AUC measurements for EPE, using 14 mm threshold and prostatectomy specimen as a reference

Extra-prostatic extension 14 mm threshold	T2WI (95%CI)	DWI (95%CI)	ADC (95%CI)	DCE (95%CI)
Sensitivity	65% (43.3–81.9)	65% (43.3–81.9)	45% (25.8%−65.8%)	90% (69.9%−97.2%)
Specificity	82.8% (73.5–89.3)	81.6% (72.2–88.4)	83.9% (74.8–90.2)	96.6%(90.3–98.8)
AUC	0.76 (0.63–0.88)	0.76 (0.61–0.88)	0.66 (0.48–0.82)	0.93 (0.86–1.00)
p-value*	0.002	0.003	< 0.001	–

*P values represent the statistical comparison of the AUC between T2WI, DWI, and ADC relative to the DCE.

*ADC* Apparent Diffusion Coefficient, *AUC* Area Under the Curve, *DCE* Dynamic Contrast Enhancement, *DWI* Diffusion Weighted Imaging, *T2WI* T2 Weighted Imaging.

**Fig. 4:**
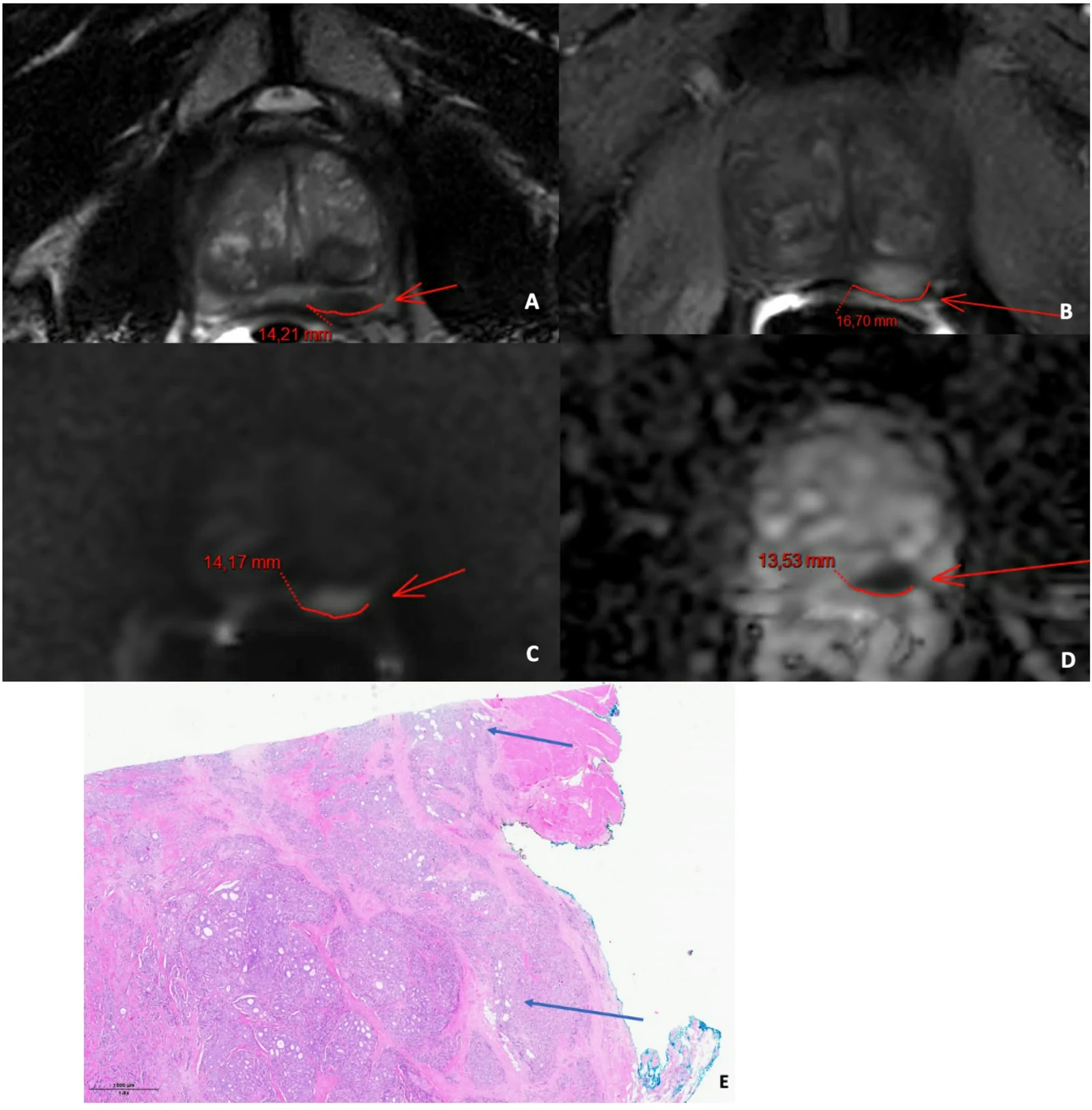
T2WI (**A**), DWI (**C**), ADC map (**D**), show a capsular contact > 10 mm and < 15 mm thresholds but > 15 mm on DCE (**B**), represented with red arrows. EPE is positive on pathology specimen (**E**): blues arrows on pathology indicate tumoral glandular proliferation extending beyond the prostatic capsule, consistent with EPE; ADC Apparent Diffusion Coefficient; DCE Dynamic Contrast Enhancement; DWI Diffusion Weighting Imaging; EPE Extra-Prostatic Extension; T2WI T2 Weighted-Imaging

Using the LCC-based method for EPE prediction, the DCE sequence outperformed T2WI, DWI, and ADC map across all tested thresholds. At a 10 mm threshold, DCE demonstrated the highest diagnostic performance with a sensitivity of 90.1% (95% CI: 68.3–98.8), specificity of 80.7% (68.1–90), and an AUC of 0.85 (0.77–0.94). At a 15 mm threshold, DCE also demonstrated better results, with a sensitivity reaching 85% (62.1–96.8), specificity, 94.7% (85.4–98.9) and AUC, 0.90 (0.81–0.94) over the other sequences (Table 3, illustrated in Fig. 4).

**Table 3:** Sensitivity, specificity and AUC for detection of EPE using prostatectomy specimen as reference standard, comparing four MRI sequences (T2WI, DWI, ADC, DCE) at 10 mm and 15 mm capsular contact thresholds

Threshold of LCC		T2WI (95% CI)	DWI (95% CI)	ADC (95% CI)	DCE (95% CI)
LCC : 10 mm	Sensitivity	75% (50.9–91.3)	85% (62.1–96.8)	61.1% (35.7–82.7)	90.1% (68.3–98.8)
	Specificity	50.9% (37.1–64.6)	54.9% (40.3–68.9)	50.9% (36.8–64.9)	80.7% (68.1–90)
	AUC	0.63 (0.51–0.75)	0.70 (0.59–0.81)	0.56 (0.43–0.69)	0.85 (0.77–0.94)
	p-value*	< 0.001	0.006	< 0.001	–
LCC: 15 mm	Sensitivity	55% (31.5–76.9)	65% (40.8–84.6)	50% (26–74)	85% (62.1–96.8)
	Specificity	76.4% (63–86.8)	72.5% (58.3–84.1)	75.5% (61.7–86.2)	94.7% (85.4–98.9)
	AUC	0.66 (0.53–0.78)	0.69 (0.56–0.81)	0.63 (0.49–0.76)	0.90 (0.81–0.94)
	p-value*	< 0.001	0.002	< 0.001	–

*p-values represent the statistical comparison of AUC between T2WI, DWI, and ADC relative to DCE.

*ADC* Apparent Diffusion Coefficient, *AUC* Area Under the Curve, *DCE* Dynamic Contrast Enhancement, *DWI* Diffusion Weighted Imaging, *T2WI * T2 Weighted Imaging.

### LCC threshold: 10 mm vs. 15 mm

**Fig. 5:**
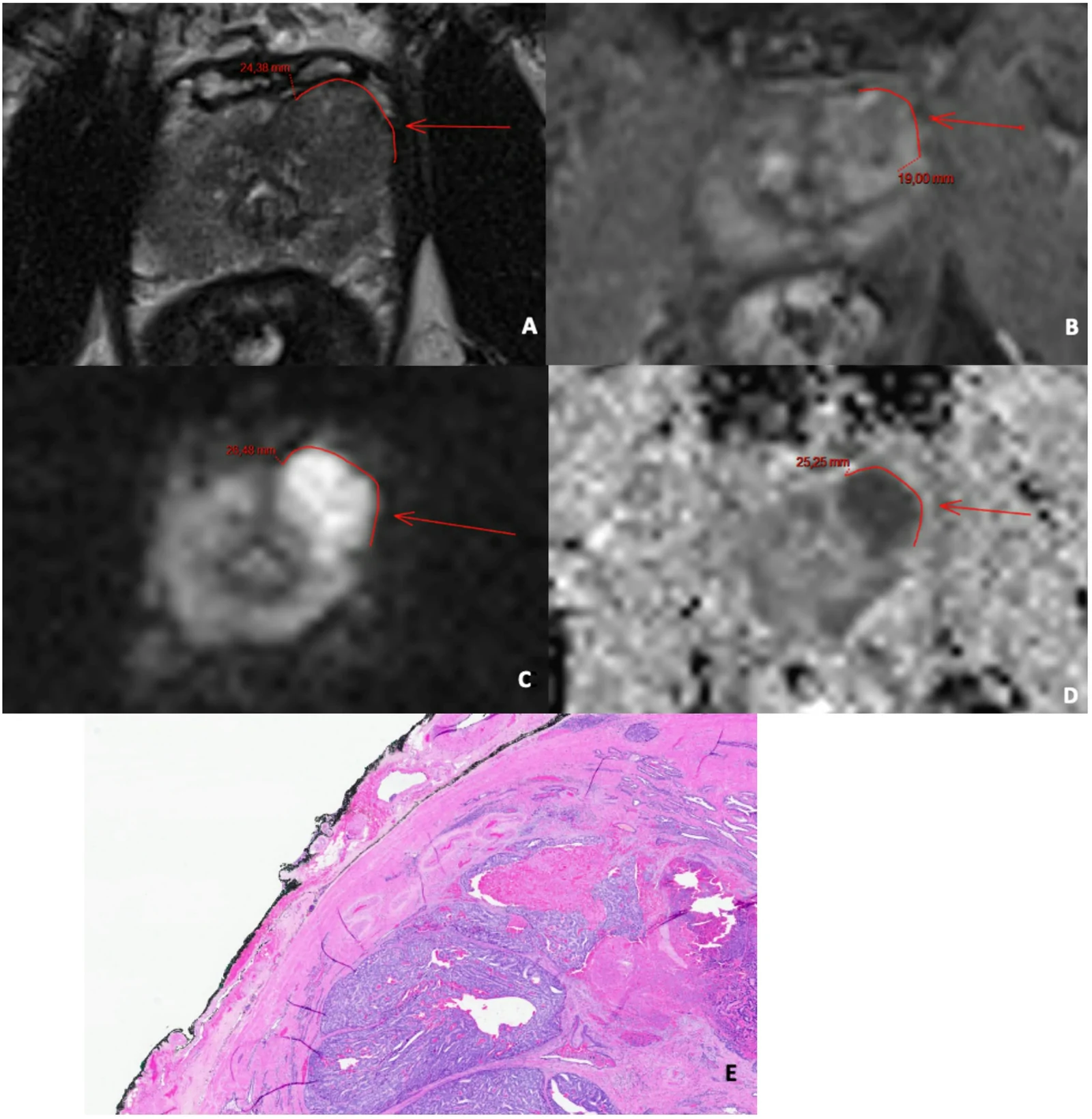
False positive case. T2WI (**A**), DCE (**B**), DWI (**C**), ADC map (**D**), show a capsular contact > 15 mm, represented with the red arrow. EPE is negative on the operative specimen (**E**); ADC Apparent Diffusion Coefficient; DCE Dynamic Contrast Enhancement; DWI Diffusion Weighting Imaging; EPE Extra-Prostatic Extension; T2WI T2 Weighted-Imaging

When increasing the LCC threshold from 10 mm to 15 mm, the diagnostic performance of DCE improved in terms of specificity and AUC. At 10 mm, DCE showed a sensitivity of 90.1% (68.3–98.8), specificity of 80.7% (68.1–90) and an AUC of 0.85 (0.77–0.94). At 15 mm on DCE, sensitivity slightly decreased to 85% (62.1–96.8), while specificity remained high at 94.7% (85.4–98.9) and the AUC was 0.90 (0.81–0.94). With the threshold increased to 15 mm, DCE maintained the highest overall diagnostic performance, with AUC values remaining stable despite the threshold change. Differences in AUC between DCE and other sequences were statistically significant (*p* < 0.001 for T2WI and ADC; *p* = 0.002 for DWI, Table 2, illustrated in Figs. 4 and 5).

### Optimal threshold of LCC to predict EPE

Using the DCE sequence, the 10 mm threshold resulted in approximately 2 false-negative and 16–17 false-positive cases, whereas the 15 mm threshold resulted in approximately 3 false-negative and 4–5 false-positive cases. The proposed 14 mm threshold provided the most favorable balance, with approximately 2 false-negative and 3 false-positive cases, corresponding to the highest overall diagnostic accuracy.

### Diagnostic performance of MaxRADD and optimal threshold assessment

Receiver operating characteristic (ROC) analysis was performed to evaluate the ability of MaxRADD to predict EPE. The diagnostic performance of MaxRADD varied across MRI sequences. The highest discriminative performance was observed on DCE imaging, with an AUC of 0.83 (95% CI 0.70–0.95), and a threshold of 11 mm yielding a sensitivity of 65% and a specificity of 88.5%, corresponding to the optimal trade-off between sensitivity and specificity.

Consistently, on DCE imaging, the mean MaxRADD was significantly higher in lesions with EPE compared to those without EPE (12.2 ± 5.4 mm vs. 5.1 ± 6.1 mm; *p* < 0.001).

The AUC values were 0.80 (95% CI 0.66–0.93) for T2WI, 0.78 (95% CI 0.64–0.91) for DWI, and 0.74 (95% CI 0.59–0.88) for ADC. Although DCE showed the numerically highest AUC, differences in diagnostic performance between sequences were not statistically significant when compared with DCE (T2WI *p* = 0.621; DWI *p* = 0.482; ADC *p* = 0.214).

### Combined LCC/MaxRADD prediction model

A combined model including both LCC and MaxRADD was explored to assess whether the combination of these two parameters improved the prediction of EPE. This combined model demonstrated a numerically higher AUC of 0.96 (95% CI 0.91–0.99) compared with LCC alone (AUC 0.93; 95% CI 0.86–1.00), although the difference did not reach statistical significance (*p* = 0.270).

## Discussion

In this study evaluating MRI-based parameters for the prediction of EPE in prostate cancer, DCE imaging consistently outperformed T2WI, DWI, and ADC across all tested thresholds, with an optimal diagnostic performance achieved at a 14 mm LCC cutoff yielding an AUC of 0.93.

MpMRI is a reliable tool for local staging of PCa and predicting EPE risk. Boesen et al. reported a moderate to strong correlation between mpMRI signs of EPE and pathology (AUC 0.65–0.86), based on T2WI sequences [18]. Giganti et al. demonstrated that pathological ADC values, tumor volume, and biopsy Gleason score were independent predictors of EPE, incorporated into a predictive nomogram achieving sensitivity of 88% compared to T2WI alone (54%; *P* = 0.010), with an overall accuracy of 81% on external validation [19]. Li et al. showed that LCC yielded a sensitivity of 79% and specificity of 77% (AUC 0.67) for predicting EPE. Xiao et al. reported a pooled AUC of approximately 0.79, with sensitivity of 76% and specificity of 68% at a 10 mm cutoff, and sensitivity of 70% with specificity of 74% at 15 mm, though no optimal cutoff could be established due to study heterogeneity [20, 21].

The optimal LCC threshold for predicting EPE on MRI remains debated. Currently, PI-RADS guidelines recommend a 10 mm cutoff, whereas ESUR guidelines suggest 15 mm [15]. Baco et al. proposed a 20 mm threshold with diagnostic accuracy of 82%, sensitivity of 79%, and specificity of 85%, without DCE in their protocol [12]. Onay and Bakir et al. reported thresholds of 15.8 mm and 14.5 mm based on T2WI (AUC 0.73) [22]. Rosenkrantz et al. advocated for lower cutoffs of 6 mm on T2WI and 7 mm on ADC maps [23]. Regarding DCE specifically, Onay et al. found in 2019 that DCE correlated most strongly with pathological LCC, proposing a 13 mm threshold (AUC 0.72–0.73) [24]. Eurboonyanun et al. confirmed DCE as the most accurate sequence (AUC 0.797, 15 mm cutoff; sensitivity 75.86%, specificity 72.09%) [25]. Asfuroglu et al. similarly identified DCE as the most reliable sequence (AUC 0.815 for reader 1) [26]. The higher DCE performance in our study may partly reflect protocol-related differences, notably 23 continuously acquired dynamic phases, ZOOMit reduced-FOV DWI, and high-resolution triplanar T2WI, alongside consensus reading by experienced radiologists.

In comparison, while our results demonstrate similar accuracy for T2WI and ADC compared to previous works, we found higher diagnostic performance for DCE at a 14 mm threshold, achieving an AUC of 0.93, sensitivity of 90%, and specificity of 96.6%, indicating superior accuracy of DCE in predicting EPE in our cohort. However, this high AUC should be interpreted with caution given the single-center design of the study. Combining LCC with MaxRADD yielded numerically higher performance (AUC 0.96), though this difference was not statistically significant. We identified an optimal MaxRADD threshold of 11 mm on DCE (sensitivity 65%, specificity 88.5%), consistent with the previously reported cutoff of ≥ 10.7 mm [17]. MaxRADD should therefore be considered a complementary marker rather than a replacement for LCC, pending confirmation in larger prospective studies.

In 2015, a diagnostic meta-analysis including 75 studies published between 2000 and 2014 evaluated the performance of MRI for predicting EPE. Among these, 53 studies were performed with low-field MRI (from 1 to 1.5T), and 21 included DCE sequences, reporting an overall sensitivity and specificity of 57% and 91%, respectively, for predicting EPE [7]. The incorporation of DCE in prostate MRI protocols, optimization of acquisition parameters, improvements in image quality and resolution, as well as the use of 3 T MRI, may explain the higher diagnostic performance achieved in our study.

A comparative analysis between 10 mm and 15 mm LCC thresholds showed that the 10 mm cutoff (PI-RADS) yielded higher sensitivity (61.1–90.1%) but lower specificity (50.9–80.7%), while the 15 mm cutoff (ESUR) offered higher specificity (72.5–94.7%) with lower sensitivity (50–85%). Confidence intervals overlapped, indicating no statistically significant difference. Clinically, prioritizing sensitivity may be preferable, as false negatives could lead to inappropriate nerve-sparing surgery and increased risk of positive surgical margins, whereas false positives result in more conservative resections with less oncologic impact.

EPE is a key prognostic factor influencing recurrence risk and overall survival [2]. According to the 2024 Advanced Prostate Cancer Consensus Conference (APCCC), the management of advanced disease is complex and must be individualized through multidisciplinary evaluation [27]. Accurate preoperative detection is essential for treatment planning, particularly nerve-sparing surgery eligibility. DCE-based LCC measurements at a 14 mm threshold may help identify high-risk patients, reducing postoperative complications such as incontinence and erectile dysfunction. If confirmed, these findings could support updates to PI-RADS and ESUR recommendations. However, LCC is not an infallible marker of EPE, as illustrated in Fig. 5, where a lesion with an LCC greater than 15 mm was associated with no evidence of EPE on final pathology.

Regarding bpMRI versus mpMRI, several studies have demonstrated non-inferiority of bpMRI for screening and detection [28–30]. However, EPE assessment requires precise capsular evaluation, which may be more challenging on bpMRI alone, particularly when DWI is suboptimal. DCE imaging may therefore provide complementary value when bpMRI findings are equivocal or when subtle EPE is suspected. In such situations, the addition of DCE imaging could increase diagnostic confidence and improve local staging assessment.

The differences between sequences partly reflect their technical characteristics. T2WI provides excellent anatomical delineation but may miss subtle tumor infiltration. DWI and ADC offer functional information but are subject to geometric distortion, susceptibility artifacts, and lower spatial resolution. DCE generally offers higher temporal and spatial resolution with improved tumor margin visualization, facilitating more confident EPE assessment.

This study has several limitations. Its retrospective design may introduce selection bias. The sample size was relatively small, and the absence of inter-reader agreement analysis limits reproducibility assessment. Although consensus reading was intentionally used to improve lesion detection, reduce interpretative variability, and enhance consistency of quantitative measurements in this exploratory single-center cohort, this methodology limits the assessment of reproducibility and generalizability of the measurements in routine clinical practice. Future multicenter studies including independent readings with kappa or ICC analyses will therefore be necessary to validate the robustness of these findings. Lesion-level analyses may introduce intra-patient clustering effects not modeled here. The low proportion of EPE-positive lesions (20/105, 19%) may have contributed to threshold overfitting, particularly for the 14 mm DCE cutoff, despite bootstrap internal validation demonstrating stable performance. The prevalence of EPE-positive lesions in our cohort was nevertheless consistent with previously published literature [31]. External validation in larger independent cohorts remains necessary to confirm these findings.

## Conclusion

The DCE sequence provided the highest diagnostic performance for EPE prediction based on LCC, compared with T2WI, DWI, and ADC. An optimal threshold of 14 mm on DCE yielded the best accuracy. A 15 mm capsular contact threshold, as proposed by ESUR guidelines, tended to improve specificity, whereas a 10 mm threshold, in line with PI-RADS recommendations, favored sensitivity. Similarly, MaxRADD showed its highest performance on DCE with an optimal threshold of 11 mm for predicting EPE, although no statistically significant differences were observed compared with the other sequences. A combined model integrating LCC and MaxRADD showed a numerically higher AUC (0.96 vs. 0.93 for LCC alone), however without a statistically significant difference (*p* = 0.270). These findings highlight the added value of the mpMRI for local staging and treatment planning in prostate cancer.

## Data Availability

The datasets generated and analyzed during the current study are available from the corresponding author upon reasonable request. All authors had full access to the data, take responsibility for the integrity of the data, and the accuracy of the data analysis. Data were collected, verified, and analyzed according to institutional and ethical guidelines.

## Abbreviations

ADC: Apparent diffusion coefficient
AUC: Area under the curve
bpMRI: Biparametric MRI
csPCa: Clinically significantly prostate cancer
DCE: Dynamic contrast enhanced
DWI: Diffusion weighted image
EPE: Extra-prostatic extension
ESUR: European society for urogenital radiology
HIFU: High intensity focused ultrasound
ISUP: International society of urological pathology
LCC: Length of capsular contact
MaxRADD: Maximum lesion radial distance
mpMRI: Multiparametric magnetic resonance imaging
PCa: Prostate cancer
PI-RADS: Prostate imaging reporting and data system
PSA: Prostate-specific antigen
RP: Radical prostatectomy
T2WI: T2 Weighted imaging
